# Role of Vitamin D in Oral Lichen Planus: A Case Control Study

**DOI:** 10.3390/nu16162761

**Published:** 2024-08-19

**Authors:** María García-Pola, Lucía Rodríguez-Fonseca

**Affiliations:** Department of Surgery and Medical-Surgical Specialties, Faculty of Medicine and Sciences of the Health, Oviedo University, 33006 Oviedo, Spain; luciarguezfonseca@gmail.com

**Keywords:** oral lichen planus, vitamin D, vitamin D treatment, comorbidity, case-control study

## Abstract

Background: It has been reported that vitamin D deficiency may be associated with the development of oral lichen planus (OLP). Given the high prevalence of vitamin D deficiency in many countries, we sought to determine whether it constitutes a comorbidity of OLP. Methods: One hundred and twenty patients clinically and histologically diagnosed with OLP were evaluated for their serum vitamin D levels. The results were compared to results from a control series of the same number of subjects matched for age and sex. Results: Vitamin D deficiency was diagnosed in 45% (*n* = 54) of OLP patients and in 26.7% (*n* = 32) of the control group. Vitamin D supplements were being taken by 32 (26.7%) OLP patients and 15 (12.5%) subjects in the control group. A multivariate logistic regression model showed that OLP was associated with vitamin D deficiency [OR: 2.24 (1.28–3.98, *p* = 0.005)] and vitamin D supplementation [OR: 2.51 (1.25–5.22, *p* = 0.011)], even after controlling for confounding variables such as sex, age ≤60>, tobacco, and alcohol. Conclusion: The association between OLP patients and vitamin D deficiency or vitamin D supplementation suggests that further research might explore the benefits of vitamin D supplements in managing OLP patients.

## 1. Introduction

Oral lichen planus (OLP) is a chronic inflammatory oral mucosal disease with a reported prevalence of 1.01% in the general population [1]. It is more common in middle-aged individuals and women [2]. OLP is considered a potentially malignant disorder (OPMD) [3], and the current data for pooled OLP malignancy is 1.43% (95% CI = 1.09–1.80) [4].

The pathogenesis of OLP remains unclear. Several multifactorial processes with various triggers have been linked to OLP pathogenesis, such as local mechanical or chemical trauma, and normal commensal oral flora [5]. Many systemic diseases have been described in OLP patients. These include psychological disorders, endocrine disorders such as diabetes mellitus and hypothyroidism, cardiovascular diseases such as hypertension and dyslipidemia, autoimmune disorders, and viral disorders such as chronic hepatitis C [6,7,8,9,10,11,12]. Other associated factors include microelement deficiencies, such as vitamin B12, folic acid, and vitamin D [13,14,15].

Most research indicates that OLP pathogenesis involves immunological processes. T-cells trigger the apoptosis of basal cells in the oral epithelium, which stimulates a cascade of proteins through lymphoepithelial interaction, including soluble cytotoxic molecules that ultimately induce rupture of the lamina propria [16]. The first phases of OLP involve dendritic cells, particularly Langerhans cells. They primarily act through by present antigens captured from external or internal sources to T lymphocytes, especially through major histocompatibility complex molecules MHC-I and MHC-II [17,18].

Once activated, CD4 naïve T (Th0) cells are differentiated into helper (Th, CD4+) T cells and cytotoxic (CTL, CD8+) T cells. After activation of lymphocytes, there is also cellular attraction, with a secretory response of soluble molecules, cytokines, and chemokines (CC, RANTES—regulated on activation normal T cells expressed and secreted), similarly segregated by keratinocytes [17,19].

The T cells most involved in OLP pathogenesis are Th-1, Th-2, and Th-17, but Th-9, Th-22, and Th-6 cells also known to participate [20].

Th-1 cells secrete specific cytokines, including Interferon-gamma (IFN-ɤ), Interleukin (IL) IL-2, and Tumor Necrosis Factor-alfa (TNF-α). These proteins recruit monocytes and lesions to differentiate into proinflammatory M1 macrophages [20].

Th-17 cells also produce potent pro-inflammatory cytokines (interleukins, IL) and chemokinases such as IL-1, IL-2, TNF-α, IL-6; IL-17, IL-21 IL-22, IL-26, and CCl5 (RANTES), increasing the inflammatory response [19,21]. Th-9 (IL-9) synergistically strengthens the cytotoxic effects of Th-17 by inducing the matrix metalloproteinase MMP-9. MMP-9 increases the disruption of the basement membrane via its proteolytic function, causing the degradation of connective tissue matrix proteins [17].

Other cells that participate in OLP pathogenesis include Th-2 cells, which secrete IL-4, IL-5, IL-10, and IL-13, and regulatory T cells (Treg) with anti-inflammatory effects [16].

CTLs express a cell surface molecule called request for cytotoxic activity (RCA), which interacts with its receptor (RCAR) on Th cells. Activated Th cells activate CTLs by secreting IL-2 and IFN-γ, TNF-α, granzyme B, and perforins, as well as expressing FASL (ligand), which binds to FAS on keratinocytes, resulting in a caspase cascade that—in basal keratinocytes—triggers apoptosis [20].

**Figure 1:**
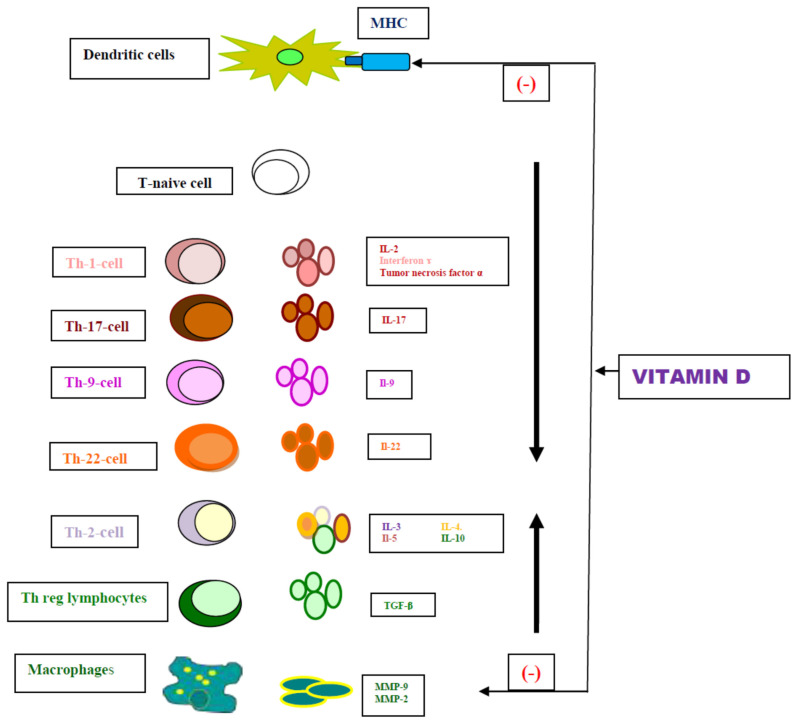
Role of vitamin D in immune pathogenesis of oral lichen planus. MHC: mayor histocompatibility complex; MMP: matrix metalloproteinases’ Th-cell: T cell helper; IL: Interleukin; TGF-β: Transforming grow factor [24,25,26,27,28,29,30,31].

Vitamin D is a steroid hormone with immunomodulation and anti-inflammatory effects [22]. This action is exerted through binding to its nuclear receptor, the vitamin D nuclear receptor (VDR), which is present in most cells and tissues, indicating its numerous physiological processes outside bone metabolism. VDR is present in keratinocytes and immune system cells such as dendritic cells, macrophages, monocytes, and T lymphocytes, among others [23]. Vitamin D has several immunological functions (Figure 1) [24,25,26,27,28,29,30,31].

Vitamin D inhibits the differentiation of monocytes into Langerhans cells, interferes in the presentation of antigens [24], and decreases the expression of MHC class II molecules [25,26].

Vitamin D promotes the differentiation of naïve T-cells into Treg and the secretion of TGF-β [27,31]. It also inhibits the differentiation of naïve T-cells into Th17 and Th1 cells [28,32]. It decreases production of Th1 lymphocytes and their function, thereby reducing the secretion of IFN-ɤ, IL-2, TNF-α, and Il-6 [31]. It also decreases the metabolism of Th-17 cells by inhibiting the production of Il-17 and Il-21 [26,29,30,33].

Vitamin D induces the production of Th-2 cytokines [30,34], which shifts T cells toward the Th-2 profile and produces anti-inflammatory Th-2 cytokines (IL-3, IL-4, IL-10, IL-19) [24,26]. Furthermore, it is also involved in direct and indirect regulation of MMP-2 and MMP-9 expression [31].

Vitamin D deficiency is defined as a 25-hydroxyvitamin D level of less than 20 ng per milliliter (50 nmol per liter) [32]. Some studies indicate that vitamin D deficiency may be a risk factor related to cancer, including oral cancer [33,34]. It has also been suggested that low vitamin D levels could increase the risk of developing OSCC from OPMDs, altering the immune response. Low levels of vitamin D are also associated with a lower survival rate in patients with OSCC [35].

Some previous studies postulated an analytical association between serum vitamin D levels in patients with OLP [36,37,38,39,40,41,42,43,44], whereas others did not [45,46,47,48]. Furthermore, a recent systematic review reported that although there were statistically significant differences between patients with OLP and a control group, the mean levels of vitamin D in patients with OLP were 26.63 ± 11.75 ng/mL, which should not be considered deficient vitamin D values [49]. Due to these contradictory results and the high prevalence of vitamin D deficiency in many countries, the purpose of this study was to evaluate serum vitamin D levels in a large sample of patients with OLP and compare the results to healthy control subjects matched for sex and age.

## 2. Materials and Methods

The present case-control study was designed according to Strengthening Reporting of Observational Studies in Epidemiology (STROBE) guidelines (Table S1) [50]. The recruitment period was based on two population groups between January 2022 and May 2024. All patients and control subjects were included consecutively in the oral lichen planus comorbidity protocol developed at the Oral Medicine Department at the University of Oviedo (Spain). The study was approved by the Ethics Committee of Asturias and performed in compliance with data protection regulations and the Declaration of Helsinki (nº 2023/140).

### 2.1. Patients

The case group consisted of 120 OLP patients over 18 years of age.

Clinical and histological inclusion criteria for OLP diagnosis were considered according to criteria from the World Health Organization (WHO) [51] and modified by van der Meij and van der Waal, and Aguirre et al. [52,53], as in previous studies [7]. Clinical criteria included the presence of bilateral, mostly symmetrical lesions, the presence of a lace-like network of slightly raised gray-white lines in a reticular pattern, and atrophic, erosive, bullous, or plaque-type lesions. Histopathological criteria included: (1) a well-defined band-like zone of cellular infiltration confined to the superficial part of the connective tissue; (2) ‘liquefactive degeneration’ in the basal layer; (3) the absence of epithelial dysplasia.

Exclusion criteria for the case group were being under 18 years old, pregnant or breastfeeding; patients treated with radiotherapy and cancer chemotherapy; oral lichenoid reaction; oral epithelial dysplasia; and OLP associated with oral cancer at the time of diagnosis. Subjects under 18 were excluded because the prevalence of childhood lichen planus has been reported to be 0.03% of the total number of cases of lichen planus [54]. Pregnant and breastfeeding women were excluded because vitamin D deficiency is very common [55,56], and oral lichenoid and dysplasia reactions were excluded to prevent confusion between OLP and oral lichenoid reactions in its nomenclature [3].

Participants were considered smokers if they were current or former smokers [57], and drinkers if their alcohol intake was at least 1 unit of alcohol per day or the equivalent in a weekend [58].

### 2.2. Control Subjects

The control group consisted of 120 patients who were examined during the same period and in the same department for reasons other than OLP. The controls were matched for age and sex to the patients with OLP.

The distribution of benign oral pathology in the control group was as follows: oral fibrous hyperplasia (*n* = 12), benign traumatic ulcer (*n* = 20), benign tumor (*n* = 18), and cheek biting (*n* = 15). The control group also included 55 patients who attended a review of their oral mucosa and whose oral mucosa was normal, without a history of LP.

Exclusion criteria from the control group were being under 18 years old, pregnant or breastfeeding, and patients treated with radiotherapy or cancer chemotherapy, as in the exclusion criteria for OLP patients.

### 2.3. Data Collection

Demographic data was collected, including sex, age, and tobacco and alcohol consumption habits. Clinical findings included OLP type according to the predominant two-thirds extension of OLP (reticular–papular and atrophic–erosive) and the number of OLP locations. The number of oral locations was 2 (by definition) or more, according to the symmetry established by the Roed Petersen scheme [51]. At the first visit, all patients were interviewed to record a case history, including medication (and any drug treatment for vitamin D deficiency), followed by an exploration of their oral cavity.

After initial exploration of the mouth, fasting venous blood was drawn from each participant to measure calcidiol-25-hydroxyvitamin D using liquid chromatography. Serum 25-OH vitamin D concentrations were analyzed by electrochemiluminescence immunoassays. Reference values are expressed in ng/mL.

### 2.4. Statistical Analysis

Data were recorded in an Excel file. Statistical analyses were performed using R (R Development Core Team, Vienna, Austria), version 4.1.3. First, a descriptive study was devised for each variable. Student’s *t*-test was used to compare the differences in average age and vitamin D level between the two groups and between each clinical form of OLP. For the analytical study, variables were considered dichotomously (presence/absence, or yes/no): mean age was classified into two categories around a breaking point of <60≥ years old; serum values for vitamin D were considered deficient below 20 ng/mL [32]; the predominant clinical form was classified as reticular–papular or atrophic–erosive; and OLP location was recorded as two locations or three-or-more locations. Pearson’s χ^2^ test was used to compare categorical parameters between groups. Results were considered statistically significant where *p* < 0.05.

Logistic regression models were used to study factors associated with the occurrence of OLP. The multivariate model was built using stepwise selection with the following variables: sex, age <60≥, tobacco use, and alcohol intake.

## 3. Results

From the initial sample of 133 patients, 13 were excluded: 11 with clinical lichenoid reaction and 2 patients with dysplasia in the histological examination.

### 3.1. General Characteristics

The sex distribution was 97 women (80.83%) and 23 men (19.17%), with a ratio of 4.2/1.

At the time of admission, the mean age of the patients was 61.06 ± 11.60 (range 20–85); for women, it was 61.69 ± 11.15 (range 20–85), and for men 58.43 ± 13.12 (range 23–80). Sixty-nine patients were >60 years old (57.5%) and the modal age was 68 years old. Demographic characteristics of the study population are shown in Table 1.

A total of 75 patients were smokers, 36 of the OLP patients and 39 of the control group, with no statistically significant differences (*p* = 0.676). Twenty-seven of the OLP patients who smoked were women and nine were men. In the control group, thirty-five of the smokers were women and four were men.

A total of 57 patients were drinkers—25 OLP patients and 32 in the control group, with no statistically significant differences (*p* = 0.289). Fourteen of the OLP drinkers were women and eleven were men. In the control group, twenty-seven drinkers were women and five were men.

The majority of OLP patients presented with ≥3 locations (70; 58.3%) and with the atrophic–erosive form (79; 65.8%).

### 3.2. Characteristics of Patients with Vitamin D Deficiency

A total of 86 (35.83%) patients had vitamin D deficiency. The prevalence of vitamin D deficiency disorder in the case group was 45% (*n* = 54), while in the control group, it was 26.7% (*n* = 32), with a crude OR of 2.25 (95% confidence interval, CI: 1.32–3.89; *p* = 0.003). The mean vitamin D serum levels in the OLP group were lower than in the control group (25.10 ±12.82 vs. 28.19 ± 14.70), with significant differences between the two groups (*p* = 0.013) (Table 1). Table 2 shows patient characteristics from the OLP and control groups with vitamin D deficiency and vitamin D intake.

The mean age of patients with vitamin D deficiency was 60.84 ± 10.52 years old in the OLP group and 62.47 ± 9.31 years old in the control group. In both OLP patients and the control group, more patients over 60 years of age (39; 72.23% and 21; 65.63%, respectively) had vitamin D deficiency.

The percentage of male patients with vitamin D deficiency was higher in the OLP group (13; 56.52%) than in the control group (4; 17.39%). The number of patients with vitamin D deficiency who smoked or were drinkers was also higher in the OLP group than in the control group.

In OLP patients with vitamin D deficiency, ≥3 locations of OLP were more common than two locations (34 vs. 20), and atrophic–erosive forms were more common than reticulo–papular forms (39 vs. 15). There were no statistically significant differences in variables related to the clinical form or number of locations between patients with a vitamin D deficiency and those with normal vitamin D levels (*p* = 0.182 and *p* = 0.352, respectively).

### 3.3. Characteristics of Patients with Vitamin D Treatment

Around a fifth of patients (47; 19.58%) used vitamin D treatments. More patients with OLP were taking vitamin D (32; 26.7%) than the controls (15; 12.5%). The crude OR associated with vitamin D treatment in OLP patients was 2.545 (CI: 1.295–5.002, *p* = 0.007).

The mean age of patients with vitamin D deficiency was 64.09 ± 11.97 years old in the OLP group and 61.85 ± 9.59 years old in the control group. There were more patients over 60 years old between OLP patients and the control group (23 at 71.88% and 10 at 66.66%, respectively).

More women than men were taking vitamin D. The percentage of women taking vitamin D was very similar in OLP patients and the control group (87.5% and 86.66%, respectively).

Regarding patients undergoing vitamin D treatment, seven (21.8%) patients from the OLP group and one (6.6%) patient from the control group were smokers. Among those taking vitamin D, seven (21.8%) patients from the OLP group and four (26.6%) patients from the control group were drinkers.

In patients with OLP taking vitamin D, the atrophic–erosive form was more prevalent (22; 68.75%), as was presenting at three or more locations (18; 56.25%). There were no statistically significant differences in the variables related to clinical form or number of locations between patients who were taking vitamin D and those who were not (*p*= 0.685 and *p* = 0.780, respectively).

### 3.4. Multivariate Analysis

The multivariate logistic regression model showed that OLP was associated with vitamin D deficiency and vitamin D supplementation, even after controlling for confounding variables, including sex, age ≤60>, tobacco, and alcohol (Table 3). The multivariate OR for vitamin D deficiency was OR = 2.24 (1.28–3.98, *p* = 0.005); for vitamin D treatment, it was OR = 2.51 (1.25–5.22, *p* = 0.011).

## 4. Discussion

The findings of this study confirmed a statistically significant association between OLP and vitamin D deficiency and vitamin D supplementation. Patients with OLP were twice as likely to exhibit vitamin D deficiency, OR: 2.24 (*p* = 0.005), or use vitamin D supplements OR: 2.51 (*p* = 0.011).

Data from epidemiological studies on OLP indicate differences in relation to sex, age, ethnicity, and geographic area, even within the same country [59]. In line with other studies, we found a higher prevalence of OLP in women [60,61,62,63] and individuals in their sixties at the time of diagnosis [57,60,64,65,66]. By contrast, other studies have indicated that it is more common in men [65] and at younger ages [61,67,68,69,70].

In the present study, patients’ smoking and alcohol habits were very similar in both groups [64,71]. However, previous studies have reported a smaller proportion of OLP patients as smokers [72,73] and more drinkers than in control groups [9,74].

Although the etiology of OLP remains uncertain, reports have described many systemic disorders linked to OLP [6,9,10,11,12,75]. In the last decade, a possible association between lower serum levels of vitamin D and LP located in the skin, mucosa [76], or in its lichen planopilaris form [77,78] has been suggested. Gholizadeh et al. also observed lower levels of vitamin D in saliva from OLP patients [46].

The mean serum vitamin D values in OLP patients in the literature range from 14.127 ng/mL [39] to 50.13 ng/mL [48], in studies performed in Egypt and Iran, respectively. The values in the present study were intermediate (25.10 ng/mL) and similar to the systematic review noted previously [49,53]. These findings suggest that the best approach to interpreting the relationship between vitamin D deficiency in OLP and the control group is to consider the relationship between the number of patients with vitamin D deficiency rather than the average from each group. This reasoning is justified by the potential compensation effect of using an average. Other definitions for the different serum values of vitamin D include *hypovitaminosis D* (*insufficient*), or vitamin D sufficiency [79], making the comparison more difficult.

Variations in vitamin D levels can also be explained because vitamin D levels depend on factors such as gender, diet, low family income, smoking, obesity, a history of diabetes or cardiovascular disease, and sun exposure [80,81]. It has also been suggested that vitamin D deficiency is more common in women and increases with age [81]. Furthermore, although the results of the present study initially indicated a higher proportion of men with OLP who had vitamin D deficiency than women (13 out of 23), multivariate analysis indicated that the risk of presenting vitamin D deficiency was independent not only of sex and age variables but also of tobacco and alcohol use.

In general terms, 37% of people worldwide have an average vitamin D level below 20 ng/mL, which is considered deficient [82]. According to geographical distribution data from the WHO, the Eastern Mediterranean region has the highest percentage of vitamin D deficiency [81].

Our findings corroborate reports from studies in China [37], India [38,44,48], Saudi Arabia [83], Egypt [39], and Croatia [36], indicating statistically significantly lower levels of mean vitamin D compared to control groups. In Iran, vitamin D levels in patients with OLP varied between 18.51 ng/mL [47] and 50.13 ng/mL [48], which complicates interpretations of sun exposure impact in the same country.

The relationship between vitamin D and OLP pathogenesis is also difficult to explain. OLP is a chronic inflammatory disease, and the majority of immune cells express VDR [84]. It is clear that certain factors may prompt a decline in vitamin D levels; for example, depression and diabetes or cardiovascular diseases are common in patients with OLP [6,8], as indicated previously. However, the relationship may also be mediated through the regulation of microRNAs that decrease VDR expression, promoting apoptosis of keratinocytes [85,86] or because one of vitamin D’s functions is to decrease interferon gene expression [87].

Dave et al.’s [60] study based on medical history determined only 8.9% of patients with OLP; in addition, 2.5% of the controls were taking vitamin D treatments in a much lower proportion than in the present study. However, similar ORs were found in the two studies, marking a difference between OLP patients and the control group (OR: 2.7 vs. 2.5).

The present study used a larger sample and aimed to shed light on the possible association between vitamin D deficiency and OLP. The role of vitamin D supplementation in improving immune diseases remains unclear [30]. A recent systematic review suggested the potential benefits of empiric vitamin D in pregnant women, children, adults over 75, and adults with prediabetes [88]. These recommendations could be extrapolated to patients with OLP who are over 75 and have prediabetes given the high prevalence [7], although always with the premise that practice guidelines do not suggest taking vitamin D routinely [89].

Regardless, some authors have suggested future research related to vitamin D therapeutic alternatives [90]. This proposal was ratified in a systematic review showing that treatment with vitamin D supplements as an adjuvant can improve OLP symptoms [91]. This approach should be considered given that El-Marssafy et al. [83] found statistically significant differences between symptomatic and asymptomatic patients, with symptomatic patients having lower vitamin D levels.

The main limitation of this study is that the control group was not from the general population despite being matched by sex and age and adjusted for other potential confounding factors such as smoking and alcohol use. Another limitation was not considering the time of year, especially lower sunlight exposure in winter months. Some of the study’s strengths included the large number of patients with histologically confirmed OLP and data collected directly from the laboratory and case history.

## 5. Conclusions

Based on the findings of the present study, patients with OLP may suffer from vitamin D deficiency and use vitamin D supplements more frequently than patients without OLP. Therefore, additional studies are recommended to explore the real benefits of vitamin D supplementation in patients with OLP.

## Figures and Tables

**Table 1:** Demographic data and vitamin D levels in patients with OLP and the control group. OLP: oral lichen planus; SD: standard deviation; ^1^: <20 ng/m. OR: Odds ratio. Statistically significant association (*p* < 0.05) *.

Variable	OLP (%, n = 120)	Control(%, n = 120)	*p*-Value
*Average years (SD)*	61.06 ± 11.60	61.06 ± 11.60	1
≤60 years	51 (42.5)	51 (42.5)	
>60 years	69 (57.5)	69 (57.5)	
*Sex*			1
Female	97 (80.03)	97 (80.03)	
Male	23 (19.17)	23 (19.17)	
*Tobacco*			0.676
Yes	36 (30)	39 (32.5)	
No	84 (70)	81 (67.5)	
*Alcohol*			0.289
Yes	25 (20.8)	32 (26.7)	
No	95 (79.1)	88 (73.3)	
*Location*			
2	50 (41.70)		
≥3	70 (58.3)		
*Clinical form*			
Reticular-papular	41 (34.2)		
Atrophic-erosive	79 (65.8)		
*Vitamin D*			
Mean (SD)	25.1019 ± 13.60	28.1951 ± 14.70	0.013 *
*Vitamin D deficiency* ^1^			0.003 *
Yes	54 (45)	32 (26.7)	
No	66 (55)	88 (73.3)	
*Vitamin D intake*			0.007 *
Yes	32 (26.7)	15 (12.5)	
No	88 (73.3)	105 (87.5)	

**Table 2:** Characteristics of OLP patients and control group with vitamin D < 20 ng/mL and vitamin D intake. Statistically significant associations (*p* < 0.05) *.

Variable		Vitamin D < 20			Vitamin D Intake	
	OLP (%, n = 54)	Control (%, n = 32)	*p* Value	OLP (%, n = 32)	Control (%, n = 15)	*p* Value
*Average age* (SD)	60.84 ± 10.52	62.47 ± 9.31		64.09 ± 11.97	61.85 ± 9.59	
	(39–85)	(44–81)		(43–83)	(39–81)	
*≤60 years>*			0.003 *			0.055
≤60	15 (27.77)	11 (34.37)		9 (28.12)	5 (33.33)	
>60	39 (72.33)	21 (65.63)		23 (71.88)	10 (66.66)	
*Sex*			0.217			0.263
Female	41(75.92)	28 (87.5)		28 (87.5)	13 (86.66)	
Male	13 (24.08)	4 (12.5)		4 (12.5)	2 (13.34)	
*Tobacco*			0.378			0.242
Yes	14 (25.92)	12 (37.5)		7 (21.8)	1 (6.6)	
No	40 (74.08)	20 (62.5)		25 (79.2)	14 (93.4)	
*Alcohol*			0.429			0.865
Yes	13 (24.07)	12 (37.5)		7 (21.8)	4 (26.6)	
No	41 (65.93)	20 (62.5)		25 (78.2)	11 (73.4)	
*Location*			0.352			0.780
2	20 (37.0)			14 (43.8)		
≥3	34 (63.0)			18 (56.2)		
*Clinical form*			0.182			0.685
Reticular–papular	15 (27.8)					
Atrophic–erosive	39 (72.2)					

**Table 3:** Univariate and multivariate analysis of the association between deficient vitamin D and treatment with vitamin D in OLP patients and the control group. Adjusted for sex, age ≤60> tobacco, and alcohol. OR: Odd statistically significant associations (*p* < 0.05) *.

Variable	OLP n (%)	Control Group n (%)	OR Univariate (CI, *p* Value)	OR Multivariate (CI, *p* Value)
*Sex*				
Female (%)	97 (50.0)	97 (50.0)	-	-
Male (%)	23 (50.0)	23 (50.0)	1.00 (0.52–1.91, *p* = 1.000)	0.90 (0.45–1.77, *p* = 0.754)
*Age*				
≤60 (%)	51 (50.0)	51 (50.0)	-	-
>60 (%)	69 (50.0)	69 (50.0)	1.00 (0.60–1.67, *p* =1.000)	0.77 (0.44–1.33, *p* = 0.344)
*Tobacco*				
No (%)	84 (50.9)	81 (49.1)	-	-
Yes (%)	36 (48.0)	39 (52.0)	0.89 (0.51–1.54, *p* = 0.676)	1.19 (0.64–2.22, *p* = 0.578
*Alcohol use*				
No (%)	95 (51.7)	88 (48.1)	-	-
Yes (%)	25 (43.9)	32 (56.1)	0.72 (0.40–1.31, *p* = 0.289)	0.62 (0.32–1.22, *p* = 0.170)
*Vitamin D <20 ng/mL*				
No (%)	88 (57.1)	66 (42.9)	-	-
Yes (%)	54 (62.8)	32 (37.2)	2,25 (1.32–3.89, *p* = 0.003) *	2.24 (1.28–3.98, *p* = 0.005) *
*Vitamin D Treatment*				
No (%)	105 (54.4)	88 (45.6)	-	
Yes (%)	32 (68.1)	15 (31.9)	2.55 (1.32–5.12, *p* = 0.007) *	2.51 (1.25–5.22, *p* = 0.011) *

## Data Availability

The data presented in this study are available upon request from the corresponding author.

## Supplementary Materials

The following supporting information can be downloaded at: https://www.mdpi.com/article/10.3390/nu16162761/s1, Table S1: STROBE Statement—Checklist of items that should be included in reports of case-control studies.

## References

[1] González-Moles M.Á., Warnakulasuriya S., González-Ruiz I., González-Ruiz L., Ayén Á., Lenouvel D., Lenouvel D., Ruiz-Ávila I., Ramos-García P. (2021). Worldwide prevalence of oral lichen planus: A systematic review and meta-analysis. Oral Dis..

[2] Le Cleach L., Chosidow O. (2012). Clinical practice. Lichen planus. N. Engl. J. Med..

[3] Warnakulasuriya S., Kujan O., Aguirre-Urizar J.M., Bagán J.V., González-Moles M.A., Kerr A.R., Lodi G., Mello F.W., Monteiro L., Ogden G.R. (2021). Oral potentially malignant disorders: A consensus report from an international seminar on nomenclature and classification, convened by the WHO Collaborating Centre for Oral Cancer. Oral Dis..

[4] González-Moles M.Á., Ramos-García P. (2024). An Evidence-Based Update on the Potential for Malignancy of Oral Lichen Planus and Related Conditions: A Systematic Review and Meta-Analysis. Cancers.

[5] DeAngelis L.M., Cirillo N., McCullough M.J. (2019). The immunopathogenesis of oral lichen planus-Is there a role for mucosal associated invariant T cells?. J. Oral Pathol. Med..

[6] De Porras-Carrique T., González-Moles M.Á., Warnakulasuriya S., Ramos-García P. (2022). Depression, anxiety, and stress in oral lichen planus: A systematic review and meta-analysis. Clin. Oral Investig..

[7] Rodríguez-Fonseca L., Llorente-Pendás S., García-Pola M. (2023). Risk of Prediabetes and Diabetes in Oral Lichen Planus: A Case-Control Study according to Current Diagnostic Criteria. Diagnostics.

[8] De Porras-Carrique T., Ramos-García P., González-Moles M.Á. (2024). Hypertension in oral lichen planus: A systematic review and meta-analysis. Oral Dis..

[9] López-Jornet P., Parra-Perez F., Pons-Fuster A. (2014). Association of autoimmune diseases with oral lichen planus: A cross-sectional, clinical study. J. Eur. Acad. Dermatol. Venereol..

[10] De Porras-Carrique T., Ramos-García P., Aguilar-Diosdado M., Warnakulasuriya S., González-Moles M.Á. (2023). Autoimmune disorders in oral lichen planus: A systematic review and meta-analysis. Oral Dis..

[11] González-Moles M.Á., de Porras-Carrique T., Ramos-García P. (2023). Association of oral lichen planus with hepatic disorders and hepatocellular carcinoma: Systematic review and meta-analysis. Med. Oral Patol. Oral Cir. Bucal..

[12] García-Pola M., Rodríguez-Fonseca L., Suárez-Fernández C., Sanjuán-Pardavila R., Seoane-Romero J., Rodríguez-López S. (2023). Bidirectional Association between Lichen Planus and Hepatitis C-An Update Systematic Review and Meta-Analysis. J. Clin. Med..

[13] Naik S.R., Gupta P., Khaitan T., Shukla A.K. (2020). Reduced levels of serum vitamin B12 in symptomatic cases of oral lichen planus: A cross-sectional study. J. Oral Biol. Craniofac. Res..

[14] Bao Z.X., Yang X.W., Shi J., Wang Y.F. (2020). The profile of hematinic deficiencies in patients with oral lichen planus: A case-control study. BMC Oral Health..

[15] Gholizadeh N., Sheykhbahaei N. (2021). Micronutrients Profile in Oral Lichen Planus: A Review Literature. Biol. Trace Elem. Res..

[16] Kurago Z.B. (2016). Etiology and pathogenesis of oral lichen planus: An overview. Oral Surg. Oral Med. Oral Pathol. Oral Radiol..

[17] Roopashree M.R., Gondhalekar R.V., Shashikanth M.C., George J., Thippeswamy S.H., Shukla A. (2010). Pathogenesis of oral lichen planus--a review. J. Oral Pathol. Med..

[18] Jungell P. (1991). Oral lichen planus. A review. Int. J. Oral Maxillofac. Surg..

[19] El-Howati A., Thornhill M.H., Colley H.E., Murdoch C. (2023). Immune mechanisms in oral lichen planus. Oral Dis..

[20] Deng X., Wang Y., Jiang L., Li J., Chen Q. (2023). Updates on immunological mechanistic insights and targeting of the oral lichen planus microenvironment. Front. Immunol..

[21] Alrashdan M.S., Cirillo N., McCullough M. (2016). Oral lichen planus: A literature review and update. Arch. Dermatol. Res..

[22] Aribi M., Mennechet F.J.D., Touil-Boukoffa C. (2023). The role of vitamin D as an immunomodulator. Front. Immunol..

[23] Athanassiou L., Kostoglou-Athanassiou I., Koutsilieris M., Shoenfeld Y. (2023). Vitamin D and Autoimmune Rheumatic Diseases. Biomolecules..

[24] Murdaca G., Tonacci A., Negrini S., Greco M., Borro M., Puppo F., Gangemi S. (2019). Emerging role of vitamin D in autoimmune diseases: An update on evidence and therapeutic implications. Autoimmun. Rev..

[25] Brożyna A.A., Slominski R.M., Nedoszytko B., Zmijewski M.A., Slominski A.T. (2022). Vitamin D Signaling in Psoriasis: Pathogenesis and Therapy. Int. J. Mol Sci..

[26] Charoenngam N., Holick M.F. (2020). Immunologic Effects of Vitamin D on Human Health and Disease. Nutrients.

[27] Sassi F., Tamone C., D’Amelio P. (2018). Vitamin, D: Nutrient, Hormone, and Immunomodulator. Nutrients.

[28] Zhang P., Xu Q., Zhu R. (2024). Vitamin D and allergic diseases. Front. Immunol..

[29] Berretta M., Quagliariello V., Bignucolo A., Facchini S., Maurea N., Di Francia R., Fiorica F., Sharifi S., Bressan S., Richter S.N. (2022). The Multiple Effects of Vitamin D against Chronic Diseases: From Reduction of Lipid Peroxidation to Updated Evidence from Clinical Studies. Antioxidants.

[30] Holick M.F., Mazzei L., García Menéndez S., Martín Giménez V.M., Al Anouti F., Manucha W. (2023). Genomic or Non-Genomic? A Question about the Pleiotropic Roles of Vitamin D in Inflammatory-Based Diseases. Nutrients.

[31] Vo H.V.T., Nguyen Y.T., Kim N., Lee H.J. (2023). Vitamin A, D, E, and K as Matrix Metalloproteinase-2/9 Regulators That Affect Expression and Enzymatic Activity. Int. J. Mol. Sci..

[32] Holick M.F. (2007). Vitamin D deficiency. N. Engl. J. Med..

[33] Patini R., Favetti Giaquinto E., Gioco G., Castagnola R., Perrotti V., Rupe C., Di Gennaro L., Nocca G., Lajolo C. (2024). Malnutrition as a Risk Factor in the Development of Oral Cancer: A Systematic Literature Review and Meta-Analyses. Nutrients.

[34] Sagar S., Raman P., Gheena S., Abilasha R., Krishnan R.P., Selvaraj J. (2022). Salivary vitamin D levels among OSCC and normal Indian patients. Bioinformation.

[35] Maturana-Ramírez A., Aitken-Saavedra J., Guevara-Benítez A.L., Espinoza-Santander I. (2022). Hypovitaminosis D, oral potentially malignant disorders, and oral squamous cell carcinoma: A systematic review. Med. Oral Patol. Oral Cir. Bucal..

[36] Družijanić A., Cigić L., Glavina A., Draganja M., Martinović D., Ković M. (2023). Serum Concentration of Vitamin D in Patients with Oral Lichen Planus. Acta Stomatol. Croat..

[37] Du J., Li R., Yu F., Yang F., Wang J., Chen Q., Wang X., Zhao B., Zhang F. (2017). Experimental study on 1,25(OH)(2) D(3) amelioration of oral lichen planus through regulating NF-kappaB signaling pathway. Oral Dis..

[38] Gupta A., Mohan R.P., Malik S., Goel S., Gupta S. (2017). Serum Vitamin D Level in Oral Lichen Planus Patients of North India- A Case-Control Study Kamarthi, N Serum Vitamin D Level in Oral Lichen Planus Patients of North India—A Case-Control Study. J. Dermatol. Res. Ther..

[39] Mahmoud S.B., Anwar M.K., Shaker O.G., El Sharkawy D.A. (2021). Possible Relation between Vitamin D and Interleukin-17 in the Pathogenesis of Lichen Planus. Dermatology.

[40] Pawar Vinaya R., Krishna S., Deepak T.A., Prarthana G.A., Vyavahare S., Jujare Rashmi D. (2022). Association of Vitamin D Serum Concentration and Oral Lichen Planus: A Randomized Controlled Clinical Trial. J. Indian Acad. Oral Med. Radiol..

[41] Sadeghi M., Zarabadipour M., Azmodeh F., Mirzadeh M., Golezari A.S. (2020). Association of serum level of 25-hydroxyvitamin D with Oral Lichen Planus. A case-control study. J. Oral Res..

[42] Tak M.M., Chalkoo A.H. (2017). Vitamin D deficiency- A possible contributing factor in the aetiopathogenesis of oral lichen planus. J. Evol. Med. Dent. Sci..

[43] Tangarpoor M., Khademi B., Mardani M., Malekzadeh M., Jaafari-Ashkavandi Z. (2023). Vitamin D serum levels in oral lichen planus and oral cancer patients. Middle East J. Cancer.

[44] Thum-Tyzo K.J., Tyzo B.J., Chałas R. (2024). Oral lichen planus among patients from Lublin Region in relation to 25-hydroxy-vitamin D3 serum level. Ann. Agric. Environ. Med..

[45] Bahramian A., Bahramian M., Mehdipour M., Falsafi P., Khodadadi S., Dabaghi Tabriz F., Deljavanghodrati M. (2018). Comparing Vitamin D Serum Levels in Patients with Oral Lichen Planus and Healthy Subjects. J. Dent..

[46] Gholizadeh N., Pirzadeh F., Mirzaii-Dizgah I., Sheykhbahaei N. (2020). Relationship between salivary vitamin D deficiency and oral lichen planus. Photodermatol. Photoimmunol. Photomed..

[47] Nosratzehi T. (2023). Serum vitamin D and antinuclear antibody level in oral lichen planus patients: A cross-sectional study. Ann. Med. Surg..

[48] Rezazadeh F., Haghighat S. (2021). Serum Vitamin Profile in Oral Lichen Planus Patients in Southwest of Iran. Biomed Res. Int..

[49] Egido-Moreno S., Valls-Roca-Umbert J., Parra-Moreno F.J., Jané-Salas E., Blanco-Carrión A., López-López J. (2024). Association of vitamin D levels and oral lichen planus. Systematic review and meta-analysis. Med. Oral Patol. Oral Cir. Bucal..

[50] Vandenbroucke J.P., von Elm E., Altman D.G., Gøtzsche P.C., Mulrow C.D., Pocock S.J., Poole C., Schlesselman J.J., Egger M., STROBE Initiative (2007). Strengthening the Reporting of Observational Studies in Epidemiology (STROBE): Explanation and elaboration. Epidemiology.

[51] Kramer I.R., Pindborg J.J., Bezroukov V., Infirri J.S. (1980). Guide to epidemiology and diagnosis of oral mucosal diseases and conditions. Community Dent. Oral Epidemiol..

[52] van der Meij E.H., van der Waal I. (2003). Lack of clinicopathologic correlation in the diagnosis of oral lichen planus based on the presently available diagnostic criteria and suggestions for modifications. J. Oral Pathol. Med..

[53] Aguirre-Urizar J.M., Alberdi-Navarro J., Lafuente-Ibáñez de Mendoza I., Marichalar-Mendia X., Martínez-Revilla B., Parra-Pérez C., Juan-Galíndez A.D., Echebarria-Goicouria M.Á. (2020). Clinicopathological and prognostic characterization of oral lichenoid disease and its main subtypes: A series of 384 cases. Med. Oral Patol. Oral Cir. Bucal..

[54] Spirito F., Dioguardi M., Caponio V.C., Ambrosino M., Lo Muzio E., Lo Muzio L. (2024). Oral lichen planus in children: A systematic review. Med. Oral Patol. Oral Cir. Bucal..

[55] Palacios C., Gonzalez L. (2014). Is vitamin D deficiency a major global public health problem?. Steroid. Biochem. Mol. Biol..

[56] Durá-Travé T., Gallinas-Victoriano F. (2023). Pregnancy, Breastfeeding, and Vitamin D. Int. J. Mol. Sci..

[57] Carbone M., Arduino P.G., Carrozzo M., Gandolfo S., Argiolas M.R., Bertolusso G., Conrotto D., Pentenero M., Broccoletti R. (2009). Course of oral lichen planus: A retrospective study of 808 northern Italian patients. Oral Dis..

[58] Tortorici S., Corrao S., Natoli G., Difalco P. (2016). Prevalence and distribution of oral mucosal non-malignant lesions in the western Sicilian population. Minerva Stomatol..

[59] Adamo D., Calabria E., Canfora F., Coppola N., Lo Muzio L., Spirito F., Giuliani M., Azzi L., Maurino V., SIPMO (Italian Society of Oral Pathology and Medicine) (2022). Where do you live? North versus Central-South differences in relation to Italian patients with oral lichen planus: A cross-sectional study from the SIPMO (Italian Society of Oral Pathology and Medicine). BMC Oral Health.

[60] Dave A., Shariff J., Philipone E. (2021). Association between oral lichen planus and systemic conditions and medications: Case-control study. Oral Dis..

[61] Eisen D. (2002). The clinical features, malignant potential, and systemic associations of oral lichen planus: A study of 723 patients. J. Am. Acad. Dermatol..

[62] Tovaru S., Parlatescu I., Gheorghe C., Tovaru M., Costache M., Sardella A. (2013). Oral lichen planus: A retrospective study of 633 patients from Bucharest, Romania. Med. Oral Patol. Oral Cir. Bucal..

[63] Silverman S., Gorsky M., Lozada-Nur F. (1985). A prospective follow-up study of 570 patients with oral lichen planus: Persistence, remission, and malignant association. Oral Surg. Oral Med. Oral Pathol..

[64] Arduino P.G., Karimi D., Tirone F., Sciannameo V., Ricceri F., Cabras M., Gambino A., Conrotto D., Salzano S., Carbone M. (2017). Evidence of earlier thyroid dysfunction in newly diagnosed oral lichen planus patients: A hint for endocrinologists. Endocr. Connect..

[65] Pitak-Arnnop P., Subbalekha K., Sirintawat N., Tangmanee C., Auychai P., Muangchan C., Sukphopetch P., Meningaud J.P., Neff A. (2022). Are oral lichen planus patients at high risk of hepatitis C? A case-control study. J. Stomatol. Oral Maxillofac. Surg..

[66] Lauritano D., Arrica M., Lucchese A., Valente M., Pannone G., Lajolo C., Ninivaggi R., Petruzzi M. (2016). Oral lichen planus clinical characteristics in Italian patients: A retrospective analysis. Head Face Med..

[67] Gümrü B. (2013). A retrospective study of 370 patients with oral lichen planus in Turkey. Med. Oral Patol. Oral Cir. Bucal..

[68] Ingafou M., Leao J.C., Porter S.R., Scully C. (2006). Oral lichen planus: A retrospective study of 690 British patients. Oral Dis..

[69] Thongprasom K., Mravak-Stipetić M., Luckprom P., Canjuga I., Biocina-Lukenda D., Vidović-Juras D., Sikora M., Brailo V., Jirawechwongsakul S. (2009). Oral lichen planus: A retrospective comparative study between Thai and Croatian patients. Acta Dermatovenerol. Croat..

[70] Xue J.L., Fan M.W., Wang S.Z., Chen X.M., Li Y., Wang L. (2005). A clinical study of 674 patients with oral lichen planus in China. J. Oral Pathol. Med..

[71] Ali A.A., Suresh C.S. (2007). Oral lichen planus in relation to transaminase levels and hepatitis C virus. J. Oral Pathol. Med..

[72] Robledo-Sierra J., Mattsson U., Jontell M. (2013). Use of systemic medication in patients with oral lichen planus—A possible association with hypothyroidism. Oral Dis..

[73] Gorsky M., Epstein J.B., Hasson-Kanfi H., Kaufman E. (2004). Smoking habits among patients diagnosed with oral lichen planus. Tob. Induc. Dis..

[74] Pippi R., Romeo U., Santoro M., Del Vecchio A., Scully C., Petti S. (2016). Psychological disorders and oral lichen planus: Matched case-control study and literature review. Oral Dis..

[75] López-Jornet P., Camacho-Alonso F., Rodríguez-Martínes M.A. (2012). Alterations in serum lipid profile patterns in oral lichen planus: A cross-sectional study. Am. J. Clin. Dermatol..

[76] Aksu Arica D., Baykal Selcuk L., Orem A., Ural Z., Yayli S. (2020). Evaluation of serum vitamin D levels in patients with lichen. Turkderm-Turk. Arch. Dermatol. Venereol..

[77] Larkin S.C., Cantwell H.M., Imhof R.L., Torgerson R.R., Tolkachjov S.N. (2020). Lichen Planopilaris in Women: A Retrospective Review of 232 Women Seen at Mayo Clinic From 1992 to 2016. Mayo Clin. Proc..

[78] Lim S.H., Kang H., Heo Y.W., Lee W.S., Lee S. (2023). Prevalence and incidence of comorbid diseases and mortality risk associated with lichen planopilaris: A Korean nationwide population-based study. Clin. Exp. Dermatol..

[79] Wimalawansa S.J. (2024). Physiology of Vitamin D-Focusing on Disease Prevention. Nutrients.

[80] Wang T.Y., Wang H.W., Jiang M.Y. (2023). Prevalence of vitamin D deficiency and associated risk of all-cause and cause-specific mortality among middle-aged and older adults in the United States. Front. Nutr..

[81] Cui A., Zhang T., Xiao P., Fan Z., Wang H., Zhuang Y. (2023). Global and regional prevalence of vitamin D deficiency in population-based studies from 2000 to 2022: A pooled analysis of 7.9 million participants. Front. Nutr..

[82] Hilger J., Friedel A., Herr R., Rausch T., Roos F., Wahl D.A., Pierroz D.D., Weber P., Hoffmann K. (2014). A systematic review of vitamin D status in populations worldwide. Br. J. Nutr..

[83] El-Marssafy L.M., Sadek H.S., Hussein F.F., Wahdan M.A., Elkwateh W. (2022). Serum vitamin D level in healthy individuals versus patients with symptomatic and asymptomatic oral lichen planus. Cell Mol. Biol..

[84] Kongsbak M., Levring T.B., Geisler C., von Essen M.R. (2013). The vitamin d receptor and T cell function. Front. Immunol..

[85] Ge X., Xie H., Wang L., Li R., Zhang F., Xu J., Zhao B., Du J. (2021). MicroRNA-122 promotes apoptosis of keratinocytes in oral lichen planus through suppressing VDR expression. J. Cell. Mol. Med..

[86] Ge X., Wang L., Li M., Xu N., Yu F., Yang F., Li R., Zhang F., Zhao B., Du J. (2019). Vitamin D/VDR signaling inhibits LPS-induced IFN gamma and IL-1beta in Oral epithelia by regulating hypoxia-inducible factor-1alpha signaling pathway. Cell. Commun. Signal..

[87] Ge X., Wang Y., Xie H., Li R., Zhang F., Zhao B., Du J. (2022). 1,25(OH)(2) D(3) blocks IFNbeta production through regulating STING in epithelial layer of oral lichen planus. J. Cell. Mol. Med..

[88] Shah V.P., Nayfeh T., Alsawaf Y., Saadi S., Farah M., Zhu Y., Firwana M., Seisa M., Wang Z., Scragg R. (2024). A Systematic Review Supporting the Endocrine Society Clinical Practice Guidelines on Vitamin D. J. Clin. Endocrinol. Metab..

[89] Demay M.B., Pittas A.G., Bikle D.D., Diab D.L., Kiely M.E., Lazaretti-Castro M., Lips P., Mitchell D.M., Murad M.H., Powers S. (2024). Vitamin D for the Prevention of Disease: An Endocrine Society Clinical Practice Guideline. J. Clin. Endocrinol. Metab..

[90] Nazeer J., Singh S., Jayam C., Singh R., Iqubal M.A., Singh R. (2020). Assessment of the Role of Vitamin D in the Treatment of Oral Lichen Planus. J. Contemp. Dent. Pract..

[91] Saeed S., Choudhury P., Ahmad S.A., Alam T., Panigrahi R., Aziz S., Kaleem S.M., Priyadarshini S.R., Sahoo P.K., Hasan S. (2022). Vitamin D in the Treatment of Oral Lichen Planus: A Systematic Review. Biomedicines.

